# Transformative Learning of Retired High-Tech Older Male Volunteers in Taiwan: Motivation, Contribution, Change

**DOI:** 10.1093/geroni/igaf122.4220

**Published:** 2025-12-31

**Authors:** Jen-Yu Wang, Li-Hui Lin

**Affiliations:** National Chung Cheng University, Hsinchu County, Hsinchu, Taiwan (Republic of China); National Chung Cheng University, Chiayi, Chiayi, Taiwan (Republic of China)

## Abstract

As Taiwan rapidly enters a super-aged society, retired older adults are increasingly viewed as important social resources. Among them, men from the high-tech sector—who often experienced intense workloads and occupational stress—are seeking new ways to restore health and pursue meaningful engagement in later life. Guided by Mezirow’s Transformative Learning Theory, this study explores the motivations, contributions, and changes of retired high-tech older male volunteers in community-based health promotion programs. Using a qualitative design, purposive sampling recruited six retirees aged 65–75 with high-tech industry backgrounds and over three years of consistent volunteer service. Data were collected through in-depth interviews, participant observation, and document analysis. To ensure rigor, qualitative data were analyzed using Braun and Clarke’s six-step thematic analysis, allowing for systematic coding, theme development, and interpretation across multiple data sources. Three major themes emerged. First, Motivation: participants were driven by a desire for self-care, social contribution, and extending professional values. Second, Contribution: they actively supported course instruction, event planning, and organizational management by applying technical and managerial expertise. Third, Change: through reflection and peer support, volunteers overcame initial anxiety, developed a renewed identity, and shifted from health service recipients to active health promoters. Findings demonstrate that retired high-tech professionals possess strong transformative learning potential and can effectively contribute to community health initiatives. The study suggests that nonprofit and adult education organizations design practice-oriented and supportive mechanisms to sustain their participation, thereby fostering active aging and intergenerational inclusion.

